# Alternative promoter usage generates novel shorter *MAPT* mRNA transcripts in Alzheimer’s disease and progressive supranuclear palsy brains

**DOI:** 10.1038/s41598-017-12955-7

**Published:** 2017-10-03

**Authors:** Vincent Huin, Luc Buée, Hélène Behal, Julien Labreuche, Bernard Sablonnière, Claire-Marie Dhaenens

**Affiliations:** 1 Univ. Lille, Inserm, CHU Lille, UMR-S 1172 - JPArc - Centre de Recherche Jean-Pierre AUBERT Neurosciences et Cancer, F-59000 Lille, France; 2 Univ. Lille, CHU Lille, EA 2694 - Santé publique: épidémiologie et qualité des soins, Unité de Biostatistiques, F-59000 Lille, France

## Abstract

Alternative promoter usage is an important mechanism for transcriptome diversity and the regulation of gene expression. Indeed, this alternative usage may influence tissue/subcellular specificity, protein translation and function of the proteins. The existence of an alternative promoter for *MAPT* gene was considered for a long time to explain differential tissue specificity and differential response to transcription and growth factors between mRNA transcripts. The alternative promoter usage could explain partly the different tau proteins expression patterns observed in tauopathies. Here, we report on our discovery of a functional alternative promoter for *MAPT*, located upstream of the gene’s second exon (exon 1). By analyzing genome databases and brain tissue from control individuals and patients with Alzheimer’s disease or progressive supranuclear palsy, we identified novel shorter transcripts derived from this alternative promoter. These transcripts are increased in patients’ brain tissue as assessed by 5′RACE-PCR and qPCR. We suggest that these new *MAPT* isoforms can be translated into normal or amino-terminal-truncated tau proteins. We further suggest that activation of *MAPT*’s alternative promoter under pathological conditions leads to the production of truncated proteins, changes in protein localization and function, and thus neurodegeneration.

## Introduction

Along with alternative splicing and alternative transcription, alternative promoter usage (APU) is an important mechanism for transcriptome diversity and flexibility, and the control of gene expression (for a review, see^1^. Indeed, APU may modulate (i) alternative splicing, (ii) the tissue specificity^2^, regional specificity^3,4^ and/or subcellular specificity^5^ of gene expression, and (iii) gene activation during development^6^. Moreover, APU may alter the N-terminal primary structure of the expressed protein, which in turn may affect the latter’s functional activity or subcellular localization^7^. For a growing number of genes, APU has been evidenced - especially in the brain, where up to 60% of transcribed genes are concerned^4^. For instance, the existence of several different promoters has already been described for genes linked to Alzheimer’s disease (AD), such as the apolipoprotein E gene *APOE* (the main AD genetic risk factor^8^) and the presenilin-2 gene *PSEN2* (one of the catalytic moieties in the gamma secretase complex involved in amyloid precursor protein (APP) cleavage and generation of amyloid beta peptide (Aß), which accumulates outside the cell^9,10^). After Aß deposition, the second hallmark of AD is the intraneuronal aggregation of hyperphosphorylated tau proteins. Abnormal tau aggregation is also associated with other neurodegenerative diseases, referred to collectively as tauopathies.

Tau proteins are encoded by the microtubule-associated protein tau gene *(MAPT)* on chromosome 17. *MAPT* contains 15 exons, which are usually numbered from 0 to 13. *MAPT* mRNA transcripts undergo alternative splicing as a function of the developmental stage, tissue and species, with the inclusion or exclusion of six alternative exons (E2, E3, E4a, E6, E8 and E10). Exons 2, 3 and 10 are specific to the adult brain^11^, and their alternative splicing generates six different mRNA transcripts and thus six different proteins after translation (referred to as 2N3R, 1N3R, 0N3R, 2N4R, 1N4R and 0N4R). The designations 0N, 1N and 2N respectively indicate the absence of both E2 and E3, the presence of E2 but not E3, and the presence of both exons E2 and E3. The designations 3 R and 4R indicate the absence or presence of E10. Some of these isoforms aggregate with specific patterns, which define five classes of tauopathy (for a review, see^12^). These tauopathies also vary with regard to their specific clinical and neuropathological features. However, the mechanisms underlying the expression of specific *MAPT* transcripts and proteins have yet to be characterized.

To date, only one promoter (located around exon 0) has been described for *MAPT*. The existence of an additional promoter has been suggested by Andreadis^11^, with a view to explaining tissue-specific differences in *MAPT* mRNA profiles and the *MAPT* gene’s transcriptional response to transcription and growth factors. Interestingly, our study of tissue from the frontal cortex of patients with progressive supranuclear palsy (PSP) previously highlighted significant hypomethylation of a CpG site located in the CpG island shore 13kb upstream of E1^13^. This region contains several putative regulatory features, such as a CpG island, DNase I clusters, binding sites for the transcription factor CCCTC-binding factor, and the histone mark H3K27Ac. The presence of these features is compatible with the existence of an enhancer linked to another promoter downstream. Furthermore, the supposition of an alternative promoter is strengthened by reports of shorter *MAPT* mRNAs (starting from exon 1) in genome databases^14^.

Our starting hypothesis was that *MAPT* contains an alternative promoter within intron 0, and that expression of this promoter is deregulated in a context of disease. Moreover, APU might lead to the transcription of shorter transcripts, which would encode proteins with different tissue specificities and functions (as has been reported for *PSEN2* products). Shorter translation products might then be involved in the pathogenesis of tauopathies. Our present results show that a second *MAPT* promoter is located upstream of exon 1. We described its sequence and the transcription start sites for shorter transcripts (starting at exon 1). Moreover, we found that levels of these shorter transcripts are elevated in brain tissues from patients suffering from tauopathies AD or PSP.

## Results

### Bioinformatics analysis of the human *MAPT* alternative promoter

**Figure 1:**
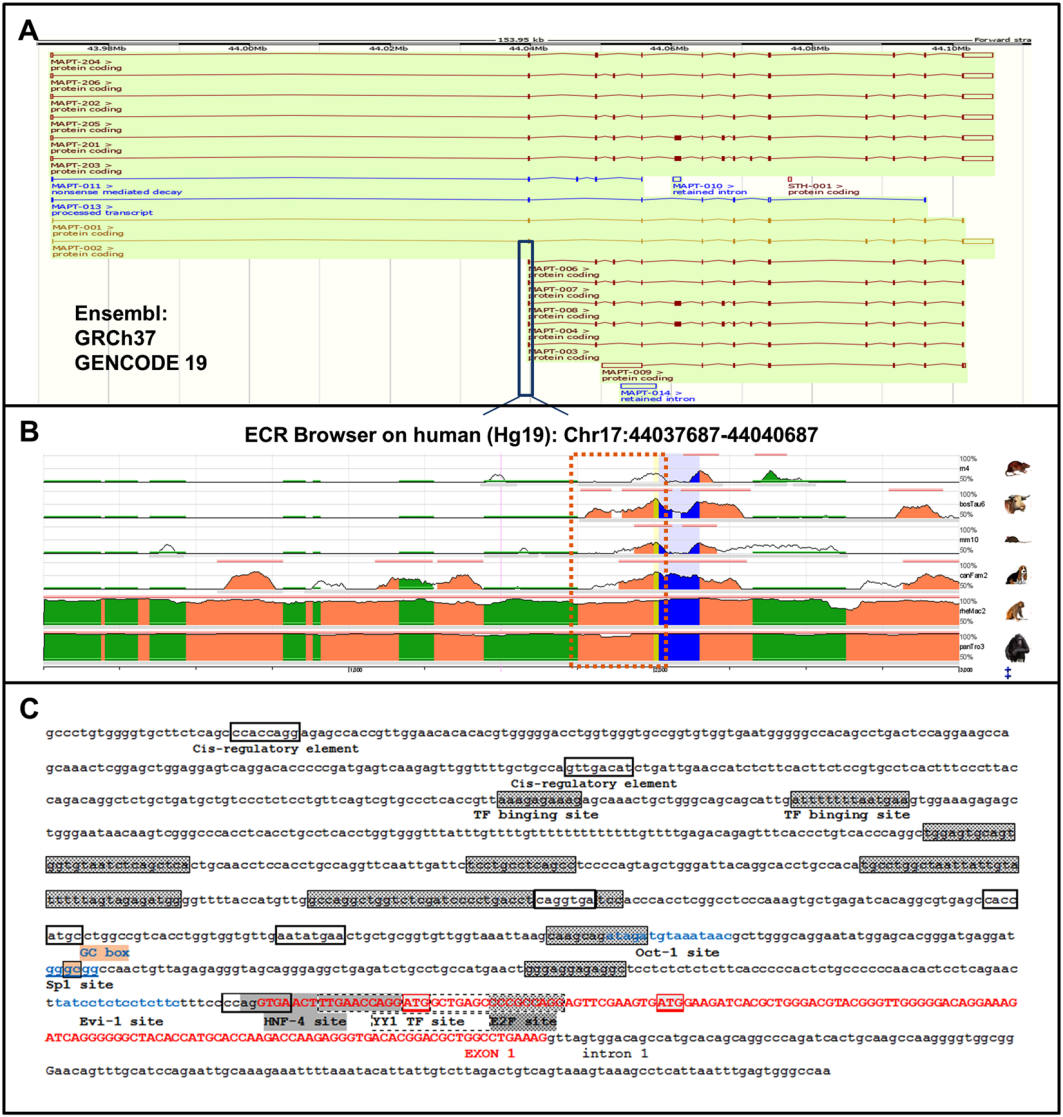
A bioinformatics analysis of the human *MAPT* gene’s alternative promoter. (**A**) The various transcripts of the *MAPT* gene reported in Ensembl: the six conventional transcripts of *MAPT* and five shorter transcripts starting in exon 1. (**B**) The exon 1 of *MAPT* and its upstream region are conserved in mammals, as shown by alignment with the human Hg19 DNA sequences of *MAPT* orthologues in various organisms, using the ECR browser (https://www.ecrbrowser.dcode.org). Exon 1’s coding sequences are shown in blue and the non-coding sequences are shown in yellow. (**C**) Sequence of the putative alternative promoter of *MAPT*. Exon 1’s sequence is depicted in red capital letters. Putative transcription factor binding sites (obtained with RSAT software and the TRANSFAC database) are marked with a white box, a gray box and in blue. The GC box is underlined in pink. Two ATG codons (corresponding to methionines 1 and 11) are framed in red.

The UCSC (genome assembly GRCh37/Hg19), Gencode V19 setting and Ensembl (GRCh37) databases contained reports of five shorter *MAPT* transcripts beginning at exon 1 (Fig. 1A). These *MAPT* transcripts contain the same coding sequence and the same alternative cassettes as the longest *MAPT* transcripts but differ in their 5′ untranslated region (UTR) sequence. This observation suggested the presence of an additional mechanism for transcriptional regulation. When looking at the human genome issue Hg19 with the ECR browser (https://www.ecrbrowser.dcode.org), we found that the region of the *MAPT* gene encompassing the 5′ intronic region upstream of exon 1 and then exon 1 itself is conserved among mammals (Fig. 1B). Moreover, the Ensembl database revealed additional transcription start sites throughout the gene but especially in exon 1. Initiation of transcription at several sites is a characteristic of TATA-less promoters and a sequence analysis did not identify any TATA or CAAT boxes for exon 0 or exon 1. Analysis with the Gene Promoter Miner server (Institute of Bioinformatics, National Chiao Tung University, Hsin-Chu, Taiwan) (http://gpminer.mbc.nctu.edu.tw/about.htm) revealed a specific feature of promoters for Sp family transcription factors: a GC box 141 bp upstream of exon 1. The Promoter 2.0 Prediction server (Center for Biological Sequence Analysis, Technical University of Denmark, Lyngby, Denmark) (http://www.cbs.dtu.dk/services/Promoter/) predicted a promoter at 900 bp (score: 1.127) and 500 bp upstream of exon 1 (score: 0.53), and at the first codon of exon 1 (score: 0.55). The Neural Network Promoter Prediction server (version 2.2, Lawrence Berkeley Laboratory, Berkeley, CA, USA) (http://www.fruitfly.org/seq_tools/promoter.html) indicated the presence of promoters at 1382 bp (score: 0.84), 568 bp (score: 0.95) and 153 bp (score: 0.92) upstream of exon 1. Lastly, the Regulatory Sequence Analysis Tools (Metazoa, Plateforme ABIMS Roscoff, France) (http://rsat.eu), Nsite (version 5.2013, Softberry, Inc., Mount Kisco, NY, USA) (http://www.softberry.com/berry.phtml?topic = nsite&group = help&subgroup = promoter) and P-Match-1.0 (Helmholtz Centre for Infection Research; TRANSFAC/BIOBASE GmbH, Braunschweig, Germany) (http://gene-regulation.com/pub/programs.html) software tools predicted a large number of *cis*-regulatory elements and transcription factor binding sites (e.g. YY1 and E2F) in the 2000 bp upstream of exon 1 (Fig. 1C).

### Functional assay of promoter B (upstream exon 1)

**Figure 2:**
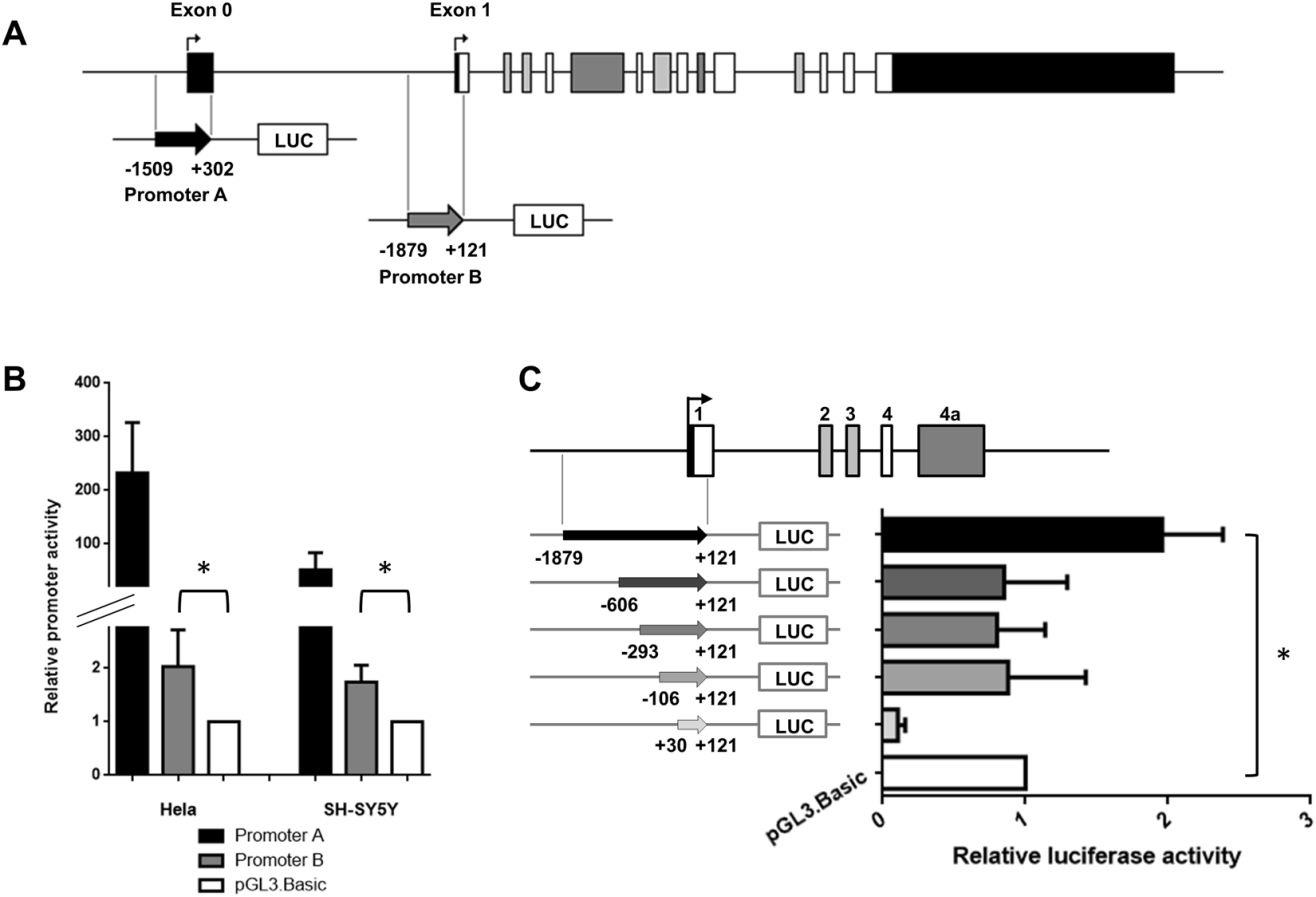
Activity of the *MAPT* promoter B in a luciferase reporter assay. (**A**) Schematic representations of the *MAPT* gene and the promoter A and B constructs. The numbering of the constructs’ 5′ and 3′ ends corresponds to the distance from the first nucleotide of exon 0 (for promoter A) or the first nucleotide of exon 1 for promoter B (Nm_005910.5; GRCh37/Hg19). (**B**) Promoter B’s activity in a luciferase reporter assay in Hela and SH-SY5Y cells is higher than that of the empty vector control (pGL3-Basic). The pGL3-promoter A construct was used as a positive control. Each experiment was repeated at least four times with triplicate samples. (**C**) Deletion analysis of the *MAPT* promoter B. Left: A schematic representation of the deletion constructs. Right: The deletion constructs’ promoter activity in a luciferase reporter assay. The B-606, B-293, B-106 and B+30 vectors had no promoter activity. Each experiment was repeated five times with triplicate samples. *p < 0.05 calculated using one sample Student test compared to the reference value of 1.

In order to demonstrate the existence of an alternative promoter, we tested the ability of a 2 kb fragment of the genomic region upstream of *MAPT* exon 1 (promoter B) to drive the expression of the luciferase gene in transiently transfected HeLa and SH-SY5Y cells (Fig. 2A). Promoter B’s activity was compared with that obtained with an empty pGL3-basic vector. In HeLa and SH-SY5Y cells, use of the promoter B construct was associated with a statistically significant, two-fold increase in luciferase gene expression (*p* = 0.028 and *p* = 0.019 vs. the empty vector for Hela and SH-SY5Y, respectively) – thus confirming promoter B’s functionality (Fig. 2B). As expected, the pGL3-promoter A construct (used as a positive control in our transient transfection studies) displayed a high level of luciferase activity (Fig. 2B).

To define the minimal essential region for the promoter B, we generated constructs with different 5′ deletions of the promoter B-1879 vector and tested them in a HeLa cell luciferase activity. Promoter B’s functional activity was lost in the B-606, B-293, B-106 and B + 30 vectors (Fig. 2C). Hence, the results of this deletion analysis did not enable us to define a classical core for promoter B – suggesting that this alternative promoter has a complex regulatory mechanism and/or structure.

### 5′RACE-PCR and characterization of the shorter transcripts

**Figure 3:**
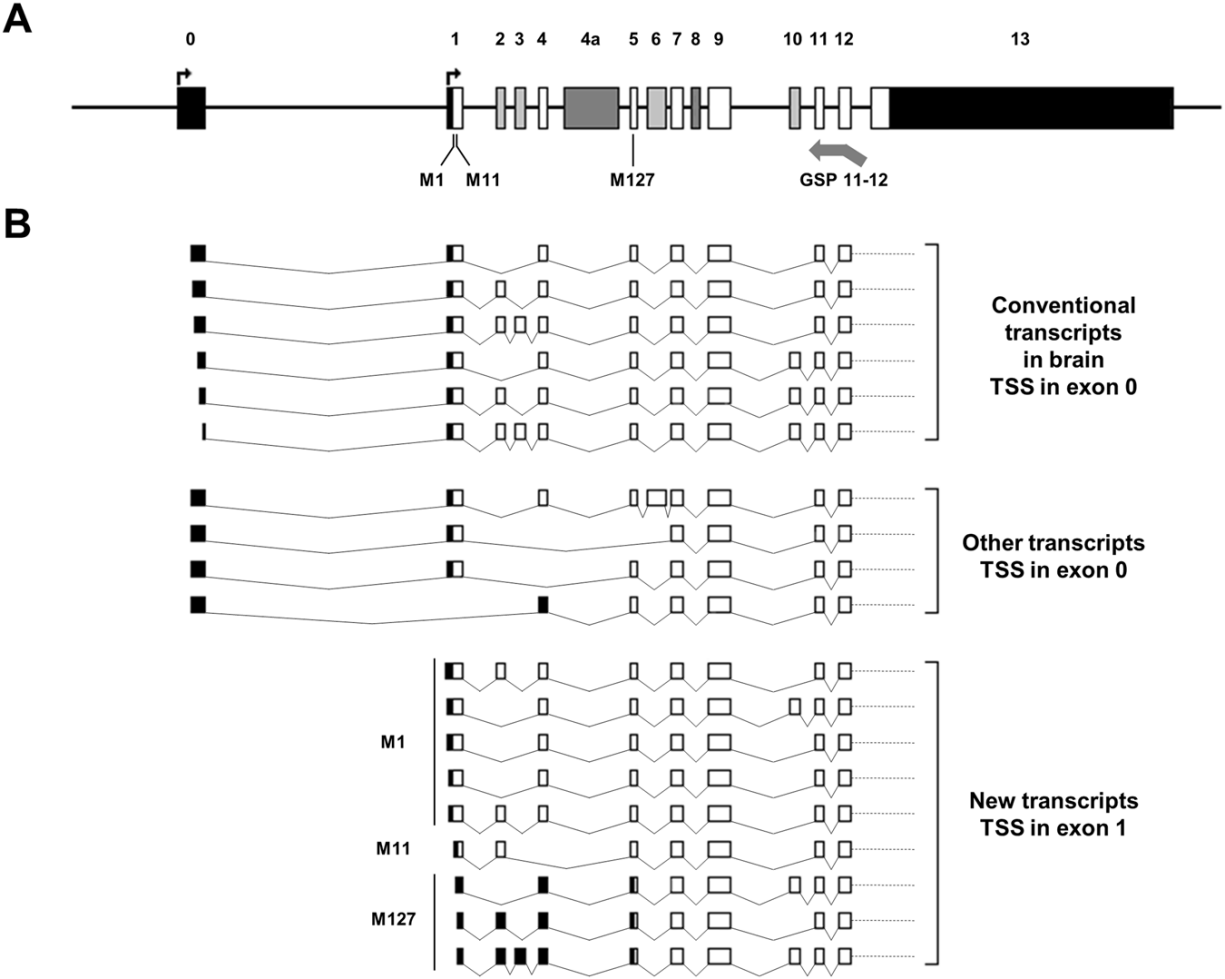
*MAPT* transcripts with different transcription start sites in human brain. (**A**) Schematic representation of the *MAPT* gene. Exons and introns are shown as boxes and horizontal lines, respectively. Untranslated regions are indicated as black boxes, constitutive exons are marked in white, alternative exons in the brain are marked in light gray, and exons presenting alternative splicing in other tissues are marked in dark gray. The different transcription initiation sites are indicated by black perpendicular arrows. The locations of the start codon (M1), the two putative alternative start codons (M11 and M127) and the GSP11-12 (arrow) are given below the *MAPT* gene (Nm_005910.5/Np_005901; GRCh37/Hg19). (**B**) Schematic representations of the different in-frame mRNAs cloned by 5′RACE, using the GSP11-12. Exons are shown as boxes and spliced introns are depicted as lines. For new transcripts with TSSs in exon 1, the predicted start codons are depicted to the left of the transcripts.

In order to assess promoter B’s functional status in human brain tissue, we performed a Rapid amplification of cDNA 5′end (5′RACE) and cloned the shortest *MAPT* mRNA isoforms. 5′RACE amplification was performed with a probe located at the junction between *MAPT* exons 11 and 12 (Fig. 3A), and enabled us to clone 64 different cDNA transcripts. Fifty-four of these were in-frame and could potentially be translated into tau proteins. More specifically, we cloned 31 cDNAs starting from different sites in exon 0 (between −205 and −11 bp upstream of the exon’s 5′ end; Fig. 3B). Interestingly we found 14 shorter clones beginning at exon 1 in all three study pools (i.e. non-clinical controls, AD patients and PSP patients). We also cloned 6 cDNAs starting from exon 2 (Supplemental Table 1). The existence of transcripts with different 5′ termini reflected the usage of the alternative promoter B of *MAPT* gene in human brain tissue.

A bioinformatics analysis of the *MAPT* sequence revealed the presence of three ATG codons respecting the consensus Kozak sequence. The first translation initiation site is located at the 18^th^ nucleotide in exon 1 (c.1, Nm_005910.5; GRCh37/Hg19); this is the conventional initiation start site for tau proteins. The second translation initiation site is located at the 48^th^ nucleotide in exon 1 (c.31), and the third and last is located at the 6^th^ nucleotide in exon 5 (c.379). With reference to the 2N4R tau isoform containing 441 amino acids (Np_005901; GRCh37/Hg19), these three translation initiation sites correspond to methionines (Met) 1, 11 and 127, respectively. These new isoforms might subsequently be translated into normal or N-terminal-truncated tau proteins.

### Short isoforms of *MAPT* transcripts are overexpressed in AD and PSP brains

#### Elevated levels of MAPT transcripts not starting at exon 0

**Figure 4:**
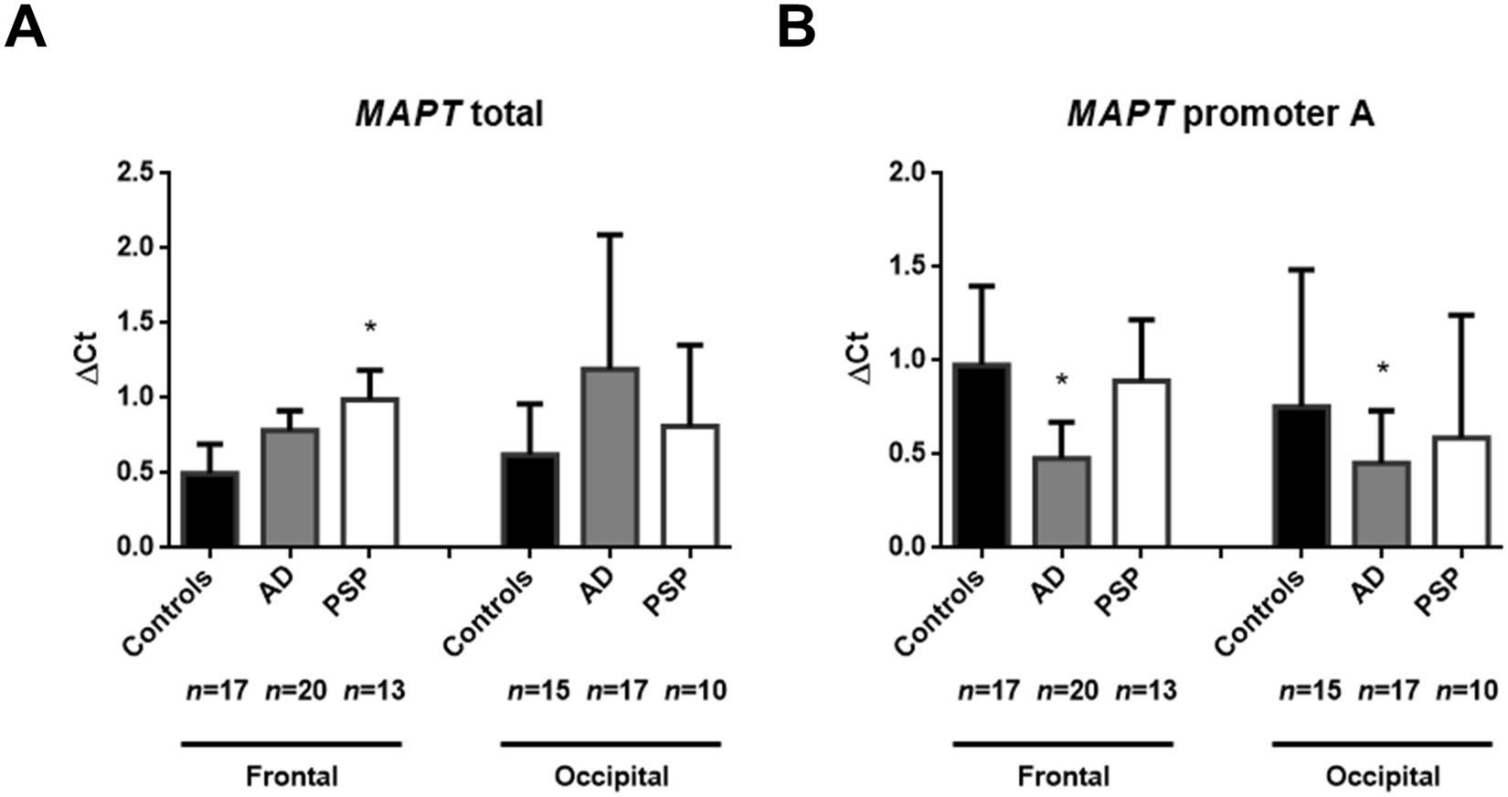
Quantitative qPCRs of the transcripts initiated from promoter B. (**A**) Quantification of all the coding *MAPT* transcripts in postmortem brain tissues, using the TaqMan® probe Hs00902194_m1. (**B**) Quantification of *MAPT* transcripts initiated from promoter A, using a custom TaqMan® probe overlapping with exon 0 and exon 1. The median [Interquartile range] level of *MAPT* expression is reported for each study group. *p < 0.05 calculated using ANOVA with posthoc Dunnett’s test AD: Alzheimer’s disease; PSP: progressive supranuclear palsy.

To measure the expression level of short *MAPT* transcripts, we used TaqMan® quantitative reverse transcriptase-PCRs (qRT-PCR) with a different set of probes. Firstly, we measured the expression level of total *MAPT* transcripts (i.e. those initiated by all putative promoters) by using a probe shared by all known coding transcripts (the exon 12/13 junction) (Fig. 4A). Secondly, the expression of transcripts initiated by the constitutive promoter A was measured with a specific probe for the exon 0/1 junction (Fig. 4B). In AD patients, *MAPT* mRNA levels were not significantly higher in either area (Fig. 4A), but were associated with low levels of transcripts initiated by promoter A in both regions (*p* = 0.0002 and *p* = 0.003, after Dunnett’s adjustment for multiple comparisons) (Fig. 4B). In samples from PSP patients, we observed significantly higher levels of total *MAPT* mRNA in tissue from the frontal cortex (*p* = 0.006) but not in the occipital cortex (which is free of neurofibrillary degeneration) (Fig. 4A). However, in frontal cortex, this elevated levels of total *MAPT* transcripts was associated with normal levels of transcripts initiated by promoter A (*p* = 0.70) (Fig. 4B). The discrepancy between the overall *MAPT* mRNA expression and expression of mRNA from promoter A strongly suggests the presence of high levels of *MAPT* transcripts not starting at exon 0 in AD and PSP brains.

#### Semi-quantitative assessment of MAPT transcripts starting at exon 1

Based on the results of the 5′RACE cloning and qPCR experiments, we next sought to directly measure the expression levels of transcripts beginning at exon 1 and thus assess their putative involvement in AD and PSP. Hence, we performed two independent 5′RACE-PCRs with mRNA from 34 brain samples (13 controls, 11 AD brains and 10 PSP brains). In line with the results of the 5′RACE-PCR using gene specific primer 11-12 (GSP 11-12), we observed a large number of exon-0-containing *MAPT* transcripts of different lengths. These appeared to be the major transcripts in the brain tissue. In all patients (but not in all controls), shorter transcripts starting at exon 1 were revealed (i) by the 5′RACE-PCR using GSP1 as a faint band of around 190 bp and (ii) by the 5′RACE-PCR using GSP4 as bands of between 240-300 bp. The electrophoresis gel and its interpretation are described in detail in Supplemental Figures S1 and S2. Hence, two independent 5′RACE-PCRs confirmed that a small proportion of *MAPT* transcripts in the brain start at exon 1.

**Figure 5:**
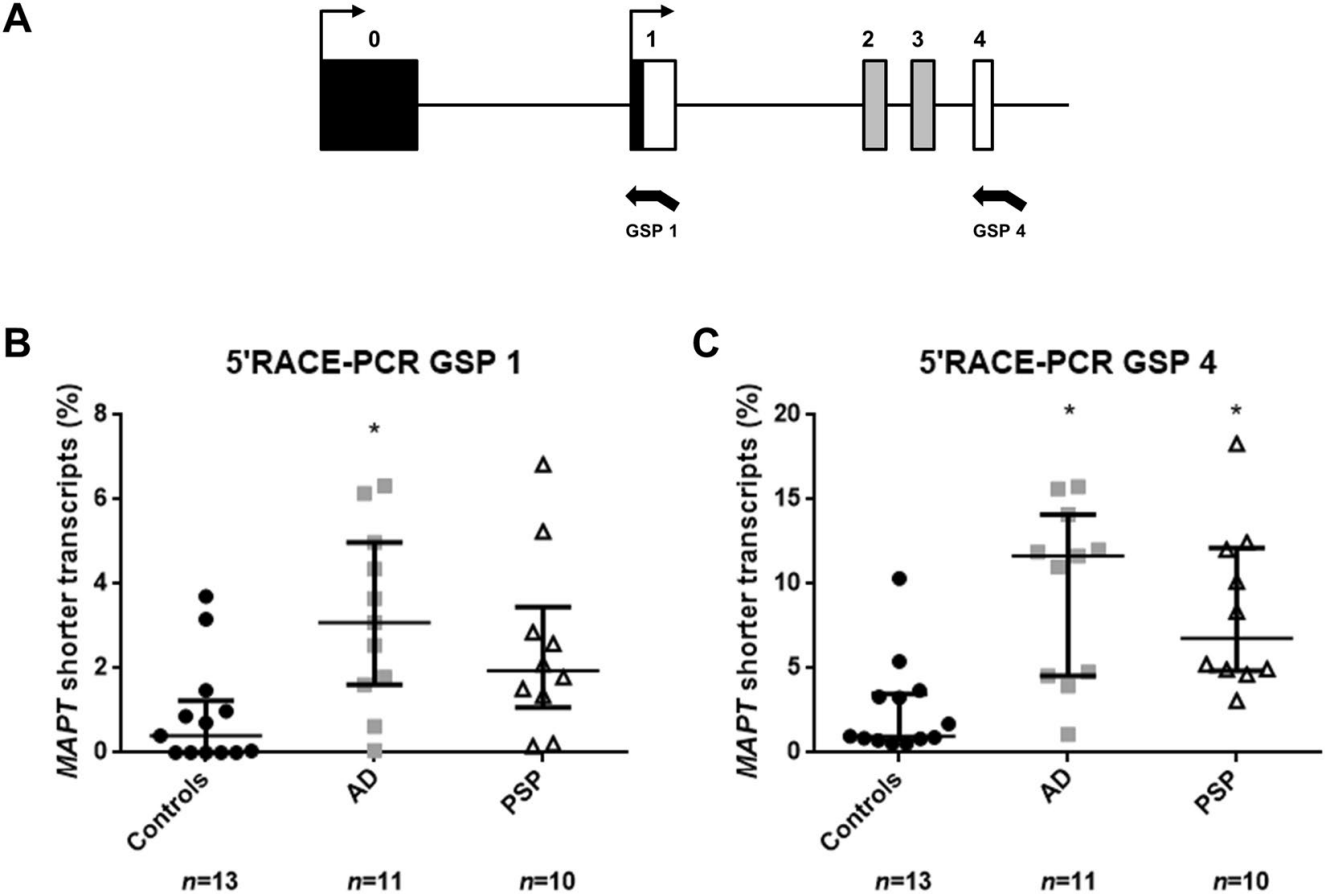
Semi-quantitative assessment of short *MAPT* transcripts using 5′RACE-PCR. (**A**) Schematic representation of the first *MAPT* exons and localization of GSP1 and GSP4. Comparison of the shorter *MAPT* transcripts in postmortem brain using 5′RACE-PCR and a GSP located in exon 1 (GSP1) (**B**) or exon 4 (GSP4) (**C**). The median [Interquartile range] level of *MAPT* expression is reported for each study group. *p < 0.05 calculated using ANOVA with posthoc Dunnett’s test. AD: Alzheimer’s disease; PSP: progressive supranuclear palsy.

Semi-quantitative assessment using Fiji ImageJ software showed significant greater levels of shorter transcripts in the frontal cortex from AD and PSP brains than in controls. This difference was observed in 5′RACE assays using GSP1 (Fig. 5A; *p* = 0.008 in the AD brain and *p* = 0.085 in the PSP brain) and GSP4 (Fig. 5B; *p* = 0.0006 in the AD brain and *p* = 0.005 in the PSP brain).

Overall, the results of the qRT-PCRs and the two independent 5′RACE-PCRs showed that although transcripts initiated by promoter B (upstream exon 1) of *MAPT* account for a small proportion of total *MAPT* transcripts, their levels are higher in AD and PSP brains than in control brains.

## Discussion

Our present results showed that like many genes expressed in the brain, *MAPT* has an alternative promoter. This promoter is located upstream of exon 1 and is active in brain tissue. Moreover, our analysis of brain tissue samples from control individuals, AD patients and PSP patients revealed (i) new, shorter transcripts issued from this alternative promoter, and (ii) additional transcripts starting from exon 0 with different transcription start sites. Lastly, we showed that *MAPT*’s alternative promoter is active in brain tissues affected by the tauopathies AD and PSP.

Although we queried three promoter prediction programs, only one predicted a promoter in exon 1. Hence, promoter prediction is a valuable tool but the outputs must be interpreted with a degree of caution. In fact, these prediction techniques are based on the accumulation of indirect markers of the presence of a promoter (such as GC, TATA and CAAT boxes). In the case of *MAPT*, it has already been reported that the constitutive promoter in exon 0 is a TATA- and CAAT-less promoter^15^. Accordingly, we did not expect the alternative promoter to contain a TATA box. We therefore focused our attention on transcription factor binding sites and elements indicating the presence of a promoter in the exon 1 region. Interestingly, we observed an accumulation of transcription factor binding sites at the start of the exon 1; these included Sp1 (a transcription factor that binds to GC-rich motifs in many promoters), E2F and YY1 (transcription factors that recruit histone-deacetylases and acetylases (for a review, see^16^). This type of element might therefore exert epigenetic control over a promoter located near to exon 1.

Although luciferase expression assays showed weak activity for promoter B, the results are consistent with the weak expression observed for the short transcripts initiated by the alternative promoter in our 5′RACE-PCR. Moreover, a weak activity has already been observed for alternative promoters, and is often associated with tissue-specific expression^17,18^. For example, the *APOE* gene (the main risk factor described for AD) has many promoters and is reportedly down-regulated by a factor of 2.13 in the AD temporal lobe - a region in which three transcriptional isoforms are expressed^8^. One of these isoforms is generated by an alternative promoter upstream of the second exon, and two transcription start sites (TSSs) are identified. These isoforms are specifically and differentially regulated in the AD temporal lobe - constituting a switch in promoter usage in healthy vs. AD brains. The *PSEN2* gene is also associated with an inherited predisposition to AD. The gene’s two promoters (P1 and P2) drive two alternative transcripts that differ with regard to their 5′ UTR^9,10^. Although most tissues express both transcripts, some tissues only express one - suggesting that TSS usage may be regulated in a tissue-specific manner^9^. Moreover, P1 and P2 are regulated by distinct transcription factors (Sp1 and Egr-1, respectively). Egr-1 can induce a partial shift in promoter usage by downregulating P1’s activity two-fold in neuronal cells. One can imagine that a similar mechanism applies to APU for *MAPT*, i.e. the activation of a secondary promoter in a particular cell type under pathological conditions. Alternatively, an enhancer sequence upstream of the promoter B (i.e. a sequence missing from our construct) might potentiate *MAPT*’s activity *in vivo*. Indeed, we recently characterized a regulatory region in intron 0 (upstream of a short CpG island and containing a CpG site that is hypomethylated in patients with PSP^13^), located 13kb upstream of exon 1. We suspect that this site influences *MAPT* expression, as has already been described for other elements (such as SNPs and long, non-coding RNAs) in intron 0^19–22^.

Our work described new shorter *MAPT* transcripts; short tau isoforms have already been attracting increasing attention in the field of neurodegenerative disorders. Indeed, Western blotting with antibodies against the C- or N-terminal end of the tau proteins revealed products that were initially considered to be degradation products or artifacts. Truncated tau proteins have been detected in patient brains and in murine disease models^23–25^, and induced a neurofibrillary pathology in the brain of transgenic rats^26,27^. It has been suggested that truncated tau may seed the aggregation of the full-length protein^28^. Interestingly, it has been shown that a 35 kDa C-terminal tau fragment lacking the N-terminus but containing four microtubule binding-repeats is over-represented in PSP and in neurodegenerative disorders in which 4R tau isoforms predominate but not in AD^29^. These observations confirm that (i) smaller tau isoforms can be expressed in various neurodegenerative diseases, and (ii) specific aggregative mechanisms may be linked to the isoform’s structural features. To date, it has been assumed that these small tau isoforms resulted from post-translational truncation at several sites within the full-length protein; however, the proteases responsible for this fragmentation have not been fully identified and characterized (for a review, see^30^. Our present results suggest that short isoforms are rare proteins resulting from an alternative transcription pattern. Indeed, we detected in-frame transcripts for which the first ATG was located in the 48^th^ position on the cDNA (corresponding to Met11 in the protein) or in exon 5 (corresponding to Met127). These isoforms have already been described^31^. By using a proteomics approach to study human brain tissue, Derisbourg *et al*. identified N-terminally truncated tau isoforms starting from Met11 and Met127. Lastly, we identified several transcripts with an atypical exon start or splicing sites that are not in frame. We cannot assume that these unusual transcripts are necessarily expressed in brain tissue. Indeed, they might be targeted by the nonsense-mediated decay pathway. It should be noted that some in-frame transcripts lacked exon 4, which is not supposedly an alternative exon in the literature.

These observations raise the question of the functional consequences of APU and alternative transcription start sites for the *MAPT* gene. In fact, APU diversifies the transcriptional regulation of a gene by modulating alternative splicing^32–37^, tissue specificity^2^, regional specificity^3,4^, subcellular specificity^5^, and/or the expression level. As suggested in the review by Davuluri *et al*.^38^, APU probably enables the mammalian genome to produce the required mRNA isoform in the right place at the right time. Our hypothesis is that under pathological conditions associated to epigenetic changes at the *MAPT* locus, a promoter switching occurs and could lead to a harmful process. Moreover, this APU could drive a specific splicing of the shorter transcripts. Indeed, it is now well established that splicing is physically and functionally coupled to the transcription and can be modified by the transcriptional rate (for a review, see^39^). The promoter B usage could promote a preferential exclusion or inclusion of exon 10 for instance, thereby altering the 3 R:4 R tau proteins ratio.

Our preliminary study is the first to show that the *MAPT* gene has two promoters: a more active constitutive promoter, plus an alternative promoter associated with elevated expression under pathological conditions. The function of the shorter transcripts initiated by this second promoter has yet to be established. However, we propose a new model in which the previously observed truncated tau isoforms are generated by APU and the use of alternative transcription start sites, rather than by post-translational modification. The present study’s main finding is the identification of an alternative promoter that could become a specific therapeutic target. A detailed understanding of the alternative promoter’s regulatory sites and factors might therefore enable specific blockage of the APU and (potentially) the neurodegenerative process.

## Patients, Materials and Methods

### Human brain tissue samples

Post-mortem brains were obtained from brain banks at university medical centers in Lille (France), Paris (France) and Geneva (Switzerland), following approval by the local institutional review board and the provision of written, informed consent by the donor’s family. All methods were performed in accordance with the relevant approved guidelines and regulations. Frontal and occipital brain regions were dissected by trained neuropathologists and then frozen at −80 °C prior to sample preparation and genetic analysis. The present study included brain tissue samples from 35 patients (22 with AD and 13 with PSP) and 18 non-clinical controls. Most of these cases have been described previously^13^.

### Extraction of DNA and RNA

DNA and RNA were extracted from 50 mg samples from the frontal lobe (Brodmann area 10) and the occipital lobe (Brodmann area 18). Genomic DNA was obtained by phenol-chloroform extraction. Total RNA was isolated using the RNeasy® Lipid Tissue Mini Kit (Qiagen, Courtaboeuf, France), according to the manufacturers’ instructions. Assessment of the RNA quality was based on the RNA integrity number (RIN) given by the Agilent Bioanalyzer 2100 and a RNA 6000 Nano Kit (Agilent, Courtaboeuf, France). All samples had a RNA concentration > 100 ng/µl and only samples with a RIN ≥ 5 were used for subsequent qRT-PCRs. Only frontal lobe samples with a RIN ≥ 7 (*n* = 34) were selected for the various 5′ rapid amplification of cDNA ends (RACE)-PCRs.

### *MAPT* alternative promoter constructs

To assess the functionality of the alternative promoter upstream of exon 1 (arbitrarily referred to here as promoter B), we first cloned a 2 kb region encompassing this putative sequence (1879 bp upstream of exon 1 and the first 121 bp of exon 1; forward primer: GCCTGTCCTGGAATTTCACATCAC; reverse primer: TGGTCTTGGTGCATGGTGTAGC). As a positive control, we cloned a 1.8 kb region encompassing the constitutive promoter upstream of exon 0 (referred to here as promoter A); 1509 bp upstream of exon 0 and the first 302 bp of exon 0; forward primer: AGGTCTTGAACTAGGATGGTGGC; reverse primer: GATAGTCGACAGAGGCGAGGAC. Both sequences were obtained from a control individual by using long-range PCR with Takara LA Taq DNA Polymerase (Clontech, Mountain View, CA, USA). To define the minimal essential region for promoter B, we prepared four sequential 5′-truncated constructs (727 bp, 414 bp, 227 bp and 91 bp in length) from the full-length sequence. After purification with the NucleoSpin® Gel and PCR Clean-up kit (Macherey Nagel, Düren, Germany), PCR products were cloned into pGL3-Basic vector (Promega, Madison, WI, USA) using the In-Fusion® HD cloning kit (Clontech). Constructs were purified using the Nucleobond® Plasmid No Lid kit (Macherey Nagel) and sequenced by GATC Biotech (Zurich, Switzerland).

### Cell culture, transfection and luciferase assays

HeLa and SH-SY5Y cells were cultured as monolayers in six-well plates using Dulbecco’s Modified Essential Medium (Invitrogen, California, USA) supplemented with 10% fetal bovine serum and 0.5% penicillin/streptomycin, at 37 °C in a humidified 5% CO_2_ incubator. Cells grown to a confluence of ~60% were transiently cotransfected with 2 μg of plasmid DNA (pGL3-promoter A, pGL3-promoter B, or pGL3-basic lacking an insert) and the pRL-TK Renilla Luciferase Control Reporter Vector as a control (test vector/co-reporter vector ratio: 10:1), using the Transporter™ 5 Transfection Reagent (Polysciences, Warrington, PA, USA). Luciferase activity was analyzed 72 h post-transfection with the Dual-Luciferase® Reporter Assay System (Promega) on a Mithras LB 940 Multimode Microplate Reader device (Berthold Technologies, Bad Wildbad, Germany). Each experiment was performed in triplicate on at least three independent culture passages. The pGL3-promoters’ activities were normalized against that the pGL3-basic vector lacking an insert.

### Quantitative RT-PCR

One µg of total RNA was used to generate cDNA by using the High-Capacity cDNA Reverse Transcription Kit (Applied Biosystems, Villebon sur Yvette, France) with Multiscribe Reverse Transcriptase and random primers. *MAPT* expression levels were determined by qRT-PCR, using TaqMan® Gene Expression Assays, TaqMan® Universal PCR Master Mix II, and uracil-N glycosylase (Life Technologies, Carlsbad, CA, USA). To quantify total *MAPT* mRNA levels, we used the Hs00902194_m1 probe located at the junction between exons 12 and 13 (i.e. within a region present in all coding transcripts). To assess the cDNA expression of transcripts from promoter A, we designed a custom qPCR TaqMan® assay with a probe overlapping exons 0 and 1. *UBC* (Hs00824723_m1) was used as a control housekeeping gene. For each sample, all qRT-PCRs were performed in triplicate on an ABI PRISM® 7900 HT instrument (Applied Biosystems), according to the manufacturer’s protocol. The comparative CT method (2^−ΔΔCT^) was used to calculate relative mRNA expression levels.

### 5′ RACE and cloning

To avoid the selection of patient-specific isoforms, we pooled the patients’ RNAs in equimolar concentrations. Only patients with a RIN≥7 were selected. Hence, the three pools corresponded respectively to 15 controls, 10 AD patients and 11 PSP patients. Rapid amplification of cDNA ends was carried out using a SMARTer™ RACE 5′/3′ kit (Clontech), according to the manufacturer’s instructions. For cDNA synthesis, 500 ng of total RNA was converted into RACE-ready first-strand cDNA. A 50 μl PCR reaction mixture was then prepared. It was composed of 2.5 μl 5′-RACE ready cDNA, 5 μl of 10X Universal Primer Mix, 1 μl of 10 μM gene-specific primer (GSP), and 41.5 μl of Master Mix (Clontech). The *MAPT*-specific-primer (GSP11-12: GATTACGCCAAGCTT**CTGGCCACCTCCTGGTTTATGATGGAT**) designed for use in a 5′RACE reaction was complementary to a specific mRNA sequence for the overlap between exons 11 and 12. Positive and negative controls were prepared according to the manufacturer’s protocols. The 5′RACE products were separated by electrophoresis on a 2% agarose gel (18 h at 50 mV). The smaller bands (corresponding to transcripts lacking exon 0) were cut out, and the DNA contained therein was extracted using the NucleoSpin® Gel and PCR Clean-up kit (Macherey Nagel). Transcripts were then cloned into a pRACE cloning vector (Clontech) with the In-Fusion® HD Cloning kit (Clontech). Plasmid DNAs were purified using the Nucleobond® Plasmid No Lid kit (Macherey Nagel) and sequenced by GATC Biotech.

### Semi-quantitative assessment of short *MAPT* transcripts using 5′RACE-PCRs

In order to select short *MAPT* mRNA isoforms, we perform two 5′RACE-PCRs using the protocol described above. The primers were GSP1 (GATTACGCCAAGCTT**CCCTCTTGGTCTTGGTGCATGGTGTAGC**) and GSP4 (GATTACGCCAAGCTT**ACCAGCAGCTTCGTCTTCCAGGCTG**), located in exons 1 and 4, respectively. The PCR products were separated on an 8% polyacrylamide gel. For each individual and each of the two 5′RACE-PCRs, we used Fiji ImageJ software to measure the intensity of the bands for the different *MAPT* transcripts^40^. For GSP1, the transcripts (starting at exon 1) were expected to be 194 bp or less in length. For GSP4, the expected lengths were 270, 357 and 444 bp, depending on whether or not the alternative exons 2 and 3 were included. The values correspond to the ratio between the area under the curve (AUC) for transcripts starting at exon 1 and the AUC for total *MAPT* transcripts (expressed as a percentage).

### Statistical analysis

The luciferase activities of the promoter B constructs were compared to the empty pGL3-basic vector (i.e. reference value of 1) using one sample Student’s *t* test. Comparisons of *MAPT* expression levels (qPCR and 5′RACE semi-quantitative analysis) between controls and each disease groups were performed using analysis of variance (ANOVA) with post-hoc Dunnett’s test; normality of residuals were checked using histogram and Shapiro-Wilk test. Statistical testing was performed at the 2-tailed α level of 0.05. Data were analyzed using the software package SAS, release 9.3 (SAS Institute, Cary, North Carolina, USA).

## Electronic supplementary material

Supplemental Figure S1 and S2 and Table S1.

Supplementary Table S1
