# Supplementary material for: Alternative promoter usage generates novel shorter MAPT mRNA transcripts in Alzheimer’s disease and progressive supranuclear palsy brains
Source: Sci Rep. 2017 Oct 3;7:12589. doi: 10.1038/s41598-017-12955-7 (PMC5626709; doi:10.1038/s41598-017-12955-7)
Supplement: Supplementary file 1 — Supplemental Figure S1 and S2 and Table S1. [file 41598_2017_12955_MOESM1_ESM.pdf]

Alternative promoter usage generates novel shorter MAPT mRNA transcripts in Alzheimer's disease and progressive supranuclear palsy brains.

Vincent Huin [1,*], Luc Buée [1], Hélène Behal [2], Julien Labreuche [2], Bernard Sablonnière [1], Claire-Marie Dhaenens [1]

[1] Univ. Lille, Inserm, CHU Lille, UMR-S 1172 - JPArc - Centre de Recherche Jean-Pierre AUBERT Neurosciences et Cancer, F-59000 Lille, France
[2] Univ. Lille, CHU Lille, EA 2694 - Santé publique : épidémiologie et qualité des soins, Unité de Biostatistiques, F-59000 Lille, France

*Corresponding author: Vincent Huin
Inserm UMR-S 1172, JPArc, rue Polonovski, F-59045, Lille, France
Phone: +33 320622075
Fax: +33 320538562
vincent.huin@inserm.fr

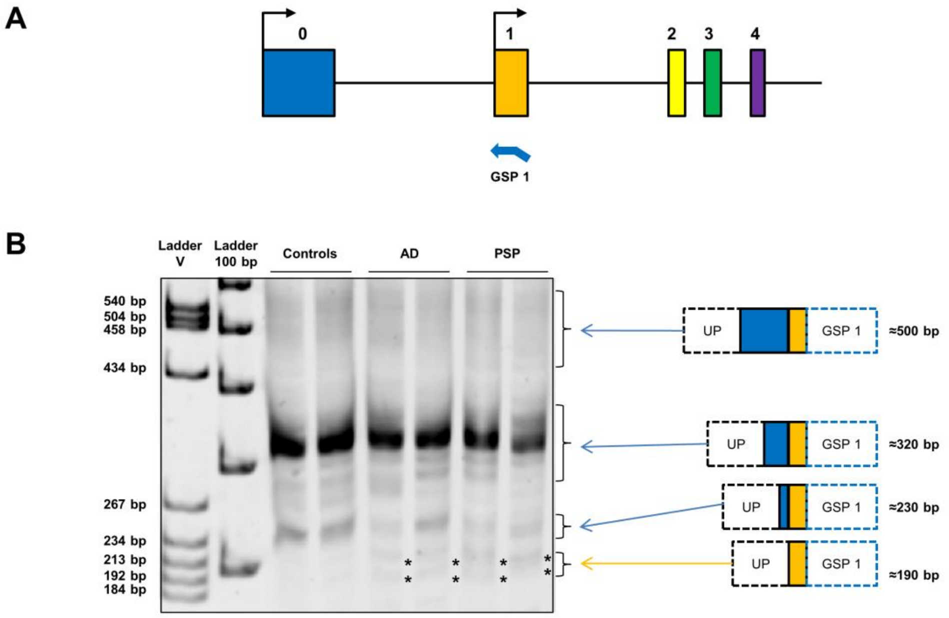

Supplemental Figure S1: 5’RACE-PCR using gene specific primer 1.
A. Schematic representation of the first *MAPT* exons and localization of GSP1. The percentage of short transcripts correspond to the ratio of the area under the curve of the transcripts starting at exon 1 (<194 bp) on the set of all the *MAPT* transcripts. **B.** Polyacrylamide gel electrophoresis of the products of 5’RACE-PCR and expected lengths using GSP1. UP: universal primer.

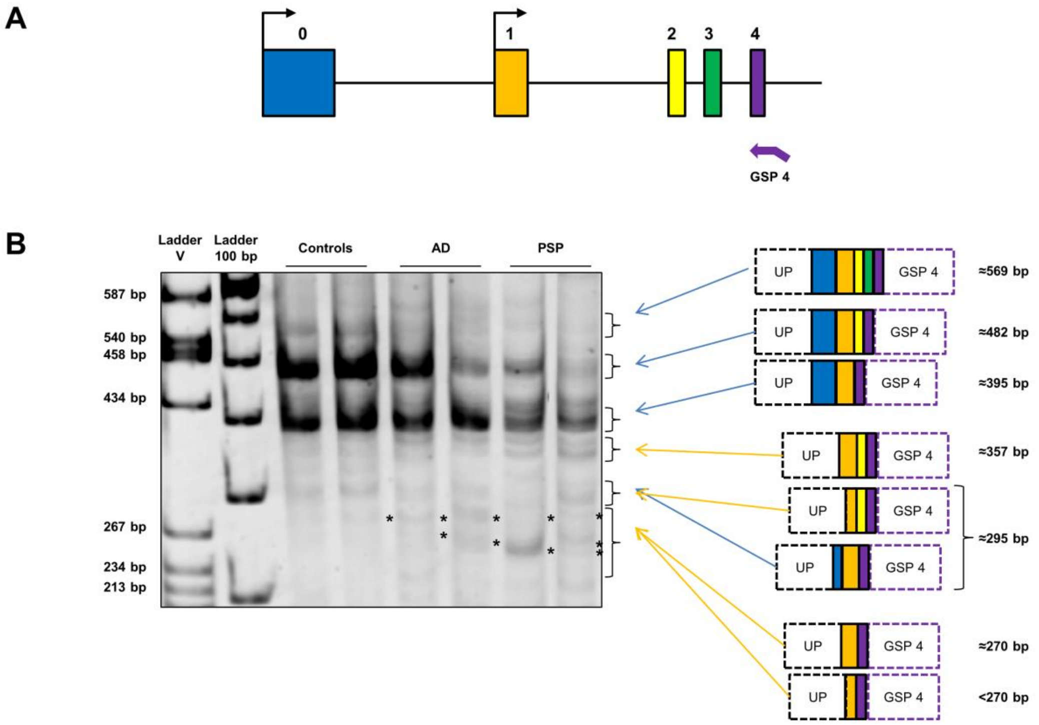

Supplemental Figure S2: 5'RACE-PCR using GSP4.
A. Schematic representation of the first *MAPT* exons and localization of GSP1. The percentage of short transcripts correspond to the ratio of the area under the curve of the transcripts starting at exon 1 (240-300 bp) and the AUC for all *MAPT* transcripts. **B.** Polyacrylamide gel electrophoresis of the products of 5'RACE-PCR and expected lengths using GSP4. UP: universal primer.

Pool	Number of clones	TSS		included exons															First Kozak	Putative protein
		exon inton	cDNA	0	1	2	3	4	4a	5	6	7	8	9	10	11	12	13		
PSP	1	Exon 0	c.-222	+	+	+		+		+		+		+	+	+	+	?	M1	1N4R
PSP	1	Exon 0	c.-174	+	+	+	+	+		+		+		+	+	+	+	?	M1	2N4R
C	1	Exon 0	c.-172	+	+	+		+		+		+		+		+	+	?	M1	1N3R
PSP	1	Exon 0	c.-170	+				+		+		+		+		+	+	?	M127 ?	ΔΔ3R ?
PSP	1	Exon 0	c.-166	+	+			+		+		+		+	+	+	+	?	M1	0N4R
MA	1	Exon 0	c.-160	+	+			+		+		+		+	+	+	+	?	M1	0N4R
PSP	1	Exon 0	c.-160	+	+	+	+	+		+		+		+	+	+	+	?	M1	2N4R
PSP	1	Exon 0	c.-158	+	+	+		+		+		+		+		+	+	?	M1	1N3R
PSP	1	Exon 0	c.-155	+	+			+		+		+		+		+	+	?	M1	0N3R
PSP	1	Exon 0	c.-153	+	+	+		+		+		+		+		+	+	?	M1	1N3R
C	1	Exon 0	c.-153	+	+			+		+		+		+		+	+	?	M1	0N3R
PSP	1	Exon 0	c.-153	+	+			+		+	+	+		+		+	+	?	M1	0N6+3R
PSP	1	Exon 0	c.-148	+	+	+		+		+		+		+		+	+	?	M1	1N3R
C	1	Exon 0	c.-144	+	+			+		+		+		+		+	+	?	M1	0N3R
PSP	2	Exon 0	c.-143	+	+	+		+		+		+		+		+	+	?	M1	1N3R
PSP	1	Exon 0	c.-141	+	+			+		+		+		+		+	+	?	M1	0N3R
PSP	1	Exon 0	c.-134	+	+	+		+		+		+		+		+	+	?	M1	1N3R
PSP	1	Exon 0	c.-125	+	+	+		+		+		+		+	+	+	+	?	M1	1N4R
PSP	1	Exon 0	c.-113	+	+			+		+		+		+	+	+	+	?	M1	0N4R
C	1	Exon 0	c.-106	+	+					+		+		+		+	+	?	M127 ?	ΔΔ3R ?
PSP	1	Exon 0	c.-101	+	+	+		+		+		+		+	+	+	+	?	M1	1N4R
C	1	Exon 0	c.-94	+	+			+		+		+		+		+	+	?	M1	0N3R
PSP	1	Exon 0	c.-54	+	+			+		+		+		+		+	+	?	M1	0N3R
MA	1	Exon 0	c.-54	+	+			+		+		+		+		+	+	?	M1	0N3R
PSP	1	Exon 0	c.-53	+	+	+	+	+		+		+		+	+	+	+	?	M1	2N4R
C	1	Exon 0	c.-51	+	+			+		+		+		+		+	+	?	M1	0N3R
MA	1	Exon 0	c.-47	+	+			+		+		+		+		+	+	?	M1	0N3R
C	1	Exon 0	c.-38	+	+	+		+		+		+		+		+	+	?	M1	1N3R
C	1	Exon 0	c.-38	+	+			+		+		+		+		+	+	?	M1	0N3R
MA	1	Exon 0	c.-35	+	+			+		+		+		+		+	+	?	M1	0N3R
C	1	Exon 0	c.-28	+	+			+		+		+		+	+	+	+	?	M1	0N4R
C	1	Intron 0	c.-17-3		+	+		+		+		+		+		+	+	?	M1	1N3R
C	1	Intron 0	c.-17-3		+			+		+		+		+		+	+	?	M1	0N3R
C	1	Intron 0	c.-17-2		+			+		+		+		+	+	+	+	?	M1	0N4R
PSP	1	Intron 0	c.-17-2		+			+		+		+		+		+	+	?	M1	0N3R
PSP	1	Exon 1	c.-9		+			+		+		+		+		+	+	?	M1	0N3R
MA	1	Exon 1	c.-7		+	+		+		+		+		+		+	+	?	M1	1N3R
PSP	2	Exon 1	c.7		+	+				+		+		+		+	+	?	M11	Δ3R
C	1	Exon 1	c.52		+			+		+		+		+	+	+	+	?	M127	ΔΔ4R
PSP	2	Exon 1	c.75		+			+		+		+		+	+	+	+	?	M127	ΔΔ4R
PSP	1	Exon 1	c.80		+			+		+		+		+	+	+	+	?	M127	ΔΔ4R
MA	1	Exon 1	c.90		+	+		+		+		+		+		+	+	?	M127	ΔΔ3R
PSP	1	Exon 1	c.94		+	+		+		+		+		+		+	+	?	M127	ΔΔ3R
C	1	Exon 1	c.108		+	+		+		+		+		+		+	+	?	M127	ΔΔ3R
MA	1	Exon 1	c.108		+	+	+	+		+		+		+	+	+	+	?	M127	ΔΔ4R
MA	1	Exon 2	c.160			+		+		+		+		+	+	+	+	?	M127	ΔΔ4R
C	1	Exon 2	c.179			+		+		+		+		+	+	+	+	?	M127	ΔΔ4R
C	1	Exon 2	c.186			+	+	+		+		+		+		+	+	?	M127	ΔΔ3R
PSP	1	Exon 2	c.195			+	+	+		+		+		+		+	+	?	M127	ΔΔ3R
C	1	Exon 2	c.195			+		+		+		+		+		+	+	?	M127	ΔΔ3R
MA	1	Exon 2	c.201			+		+		+		+		+	+	+	+	?	M127	ΔΔ4R

Supplemental Table S1: List of the *MAPT* transcripts in human brain cloned by 5’RACE.

AD: the pool of AD patients; C: the pool of controls; M1: methionine 1; M11: methionine 11; M127, methionine 127; PSP: the pool of PSP patients; Δ: putative N-terminal truncated tau proteins lacking codons 1 to 10; ΔΔ: putative N-terminal truncated tau proteins lacking codons 1 to 126. The locations of the nucleotides on the cDNA of the *MAPT* gene correspond to the longest isoform of tau protein in brain (Nm_005910.5/Np_005901; GRCh37/Hg19).
